# Impact of COVID-19 pandemic on non-COVID patient’s management in urology: a public hospital experience in Mumbai

**DOI:** 10.1186/s12301-021-00196-0

**Published:** 2021-07-06

**Authors:** Nikhilesh A. Jibhakate, Sujata K. Patwardhan, Ajit S. Sawant, Hemant R. Pathak, Bhushan P. Patil, Hitesh Kamal

**Affiliations:** 1 Department of Urology, Seth GS Medical College and KEM Hospital, Parel, Mumbai, Maharashtra 400012 India; 2 Department of Urology, Lokmanya Tilak Medical College and General Hospital, Mumbai, Maharashtra India; 3 Department of Urology, TNMC and BYL Nair Ch. Hospital, Mumbai, Maharashtra India

**Keywords:** COVID-19, Urology, Pandemic, MCGM, Mumbai

## Abstract

**Background:**

To evaluate the impact of COVID-19 lockdown on non-COVID urological patient’s management in tertiary care urology centres.

**Methods:**

This is an observational study in which data of patients visiting the urology department of all the MCGM run tertiary care hospitals were recorded for the duration of 1 April 2020 to 31 July 2020 and were compared to data of pre-COVID-19 period of similar duration.

**Results:**

There was a decrease of 93.86% in indoor admissions of urology patients during the COVID-19 lockdown. Indoor admissions for stone disease, haematuria, malignancy accounted for 53.65%, 15.85%, 9.75%, respectively. Elective surgeries had the highest percentage decrease followed by emergency and semi-emergency procedures. There was a reduction of more than 80% in patients attending outpatient clinics. Stone disease and its consequences were the main reasons for visiting outdoor clinics (39%). A substantial number of patients presented with flank and abdominal pain (14.8%) and benign enlargement of the prostate (10.23%). Malignancy accounted for a very small number of patients visiting outdoor clinics (1.58%).

**Conclusions:**

COVID-19 pandemic has a profound impact on patient care and education in Urology. There was more than ninety percent reduction in indoor admissions, operative procedures, and outpatient clinics attendance. Once the pandemic is controlled, there will be a large number of patients seeking consultation and management for urological conditions and we should be prepared for it. Surgical training of urology residents needs to be compensated in near future. Long-term impact on urological patient outcome remains to be defined.

## Background

COVID-19 was declared as a pandemic by the WHO on 11 March 2020 [1]. As of December 2020, the total number of COVID-19 infected patients in India is more than ten million**.** Mumbai, the capital city of Maharashtra, is one of the most affected cities. Municipal Corporation of Greater Mumbai (MCGM) run tertiary care public hospitals includes King Edward Memorial Hospital (KEMH), Lokmanya Tilak Municipal General Hospital (LTMGH), and BYL Nair Charitable Hospital (Nair hospital). These have the busiest urology department in the state of Maharashtra where various urological services are provided. But since the lockdown, which was announced on 25 March 2020, these hospitals have been converted into COVID dedicated facilities to deal with the increasing number of COVID-19 patients. Urology wards were converted into medicine wards to accommodate COVID-19 patients. Since the beginning of lockdown, all elective outpatient clinics, elective indoor admissions, and elective operative procedures were suspended. As recommended by guidelines, only emergency patients were attended and emergency procedures were performed, which included patients with malignancy, haematuria, urosepsis, renal trauma, acute kidney injury, and complications of other specialities like obstetrics and gynaecology etc. [2, 3]. Despite lockdown, symptomatic patients presented in outdoor clinics and emergency departments and were attended.

Taking consideration of all the above factors, we compared the data of outdoor clinics, operation theatres, indoor admissions, during the COVID-19 pandemic with data of similar duration in pre-COVID-19 period and studied the impact of COVID-19 pandemic on non-COVID patient’s management in urology in Municipal Corporation of Greater Mumbai (MCGM) run tertiary care urology centres.

## Methods

This is an observational study carried out during the period of lockdown from 1 April 2020 to 31 July 2020. Data of patients visiting the urology department in all the MCGM run tertiary care hospitals were recorded and compared to the data of the pre-COVID-19 period of similar duration. Data included indoor admissions and their indications, elective procedures, emergency procedures, and outpatient clinic attendance.

## Results

### Impact of COVID-19 on indoor admissions

**Fig. 1:**
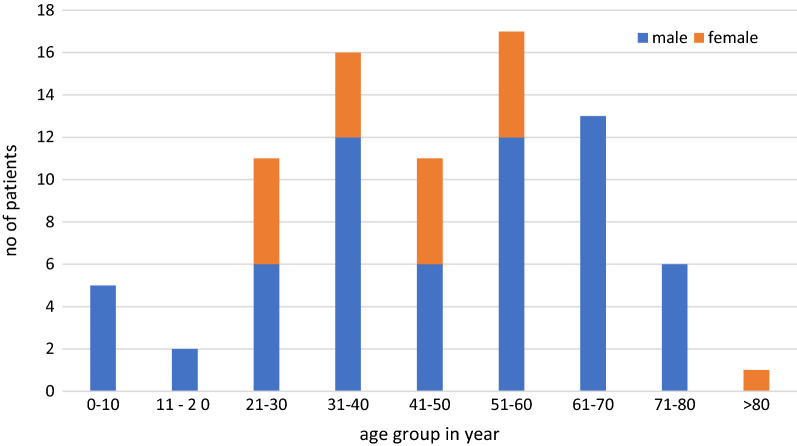
Demographic profile of indoor patients during COVID period

**Fig. 2:**
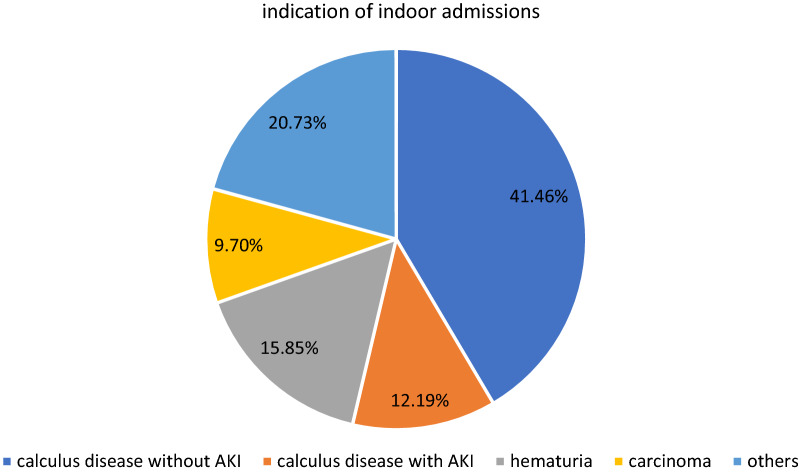
Percentage distribution according to the indication of indoor patients during COVID period

**Fig. 3:**
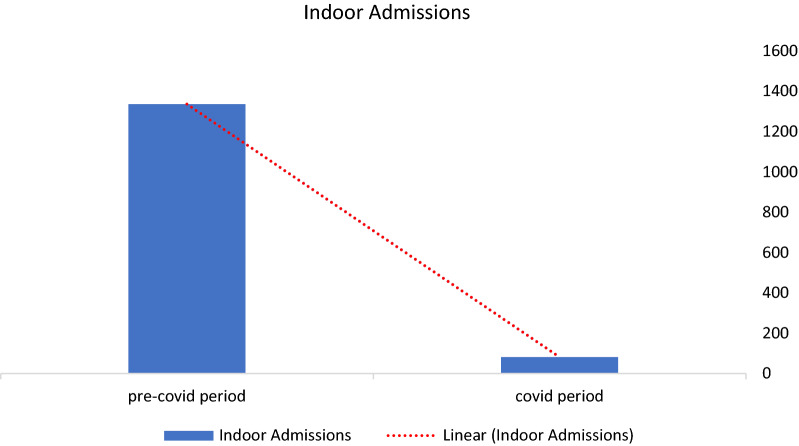
Percentage decrease in indoor admissions during COVID period

Total indoor admissions during the COVID period were 82 as compared to 1337 in the pre-COVID-19 period of the same duration. There was a decrease of 93.86% in indoor admissions. Among admitted patients in urology wards, 62 (75.6%) were male and 20 (24.4%) were female. Mostly, the patients were in the age group of 51–60 years. The most common indication of admission was symptomatic renal or ureteric calculus disease and accounted for 44 (53.65%) admissions. Haematuria was the next common indication for indoor admission accounting for 13 (15.85%) admissions. Malignancy accounted for eight admissions (9.75%) (Figs. 1, 2, 3 and Tables 1, 2).

**Table 1:** Demographic profile of indoor patients during COVID-19 lockdown

Age group	Male	Female
0–10	5	0
11–20	2	0
21–30	6	5
31–40	12	4
41–50	6	5
51–60	12	5
61–70	13	0
70–80	6	0
81 and above	0	1
Total	62	20

**Table 2:** Distribution of indoor patients according to the diagnosis

Diagnosis	No of patients
Calculus disease without AKI	34
Calculus disease with AKI	10
Urinary tract infection	4
Carcinoma bladder	5
Carcinoma penis	1
Carcinoma prostate	2
Haematuria	13
Benign enlargement of prostate	2
Emphysematous cystitis	1
Genitourinary tuberculosis	1
Radiation cystitis	2
Renal angiomyolipoma	2
BCG cystitis	1
Ureteropelvic junction obstruction	3
Emphysematous pyelonephritis	1
Total	82

### *Impact of COVID*-*19 on emergency and semi-emergency procedures*

**Fig. 4:**
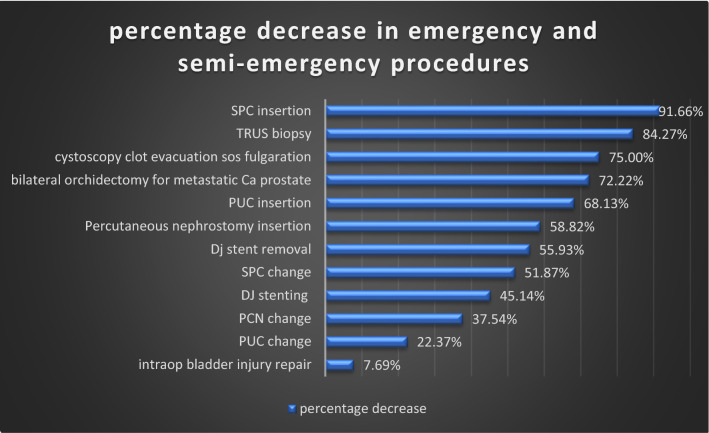
Percentage decrease in emergency and semi-emergency procedures during COVID period

There was a significant reduction in emergency and semi-emergency procedures performed during the lockdown. Procedures like cystoscopy and clot evacuation for haematuria, bilateral orchidectomy for metastatic carcinoma of the prostate, TRUS biopsy, catheter insertion had the highest cut-down, while procedures like DJ stenting, percutaneous nephrostomy insertion for AKI, infection and obstruction, DJ stent removal, and catheter change had less percentage decrease (Fig. 4 and Table 3).

**Table 3:** Comparison of emergency and semi-emergency procedures during COVID-19 lockdown and pre-lockdown period

Intervention	Pre-lockdown	Lockdown	Percentage decrease (%)
DJ stenting including exchange	175	96	45.14
Percutaneous nephrostomy insertion	68	28	58.82
DJ stent removal	177	78	55.93
TRUS biopsy	159	25	84.27
Bilateral orchidectomy	18	5	72.22
Cystoscopy with clot evacuation sos fulgaration	20	5	75
Intraop bladder injury repair	13	12	7.69
Emergency open nephrectomy for EPN	2	1	50
PUC insertion	91	29	68.13
SPC insertion	60	5	91.66
PCN change	269	168	37.54
PUC change	143	111	22.37
SPC change	133	64	51.87

### Impact of COVID-19 on elective operations

**Fig. 5:**
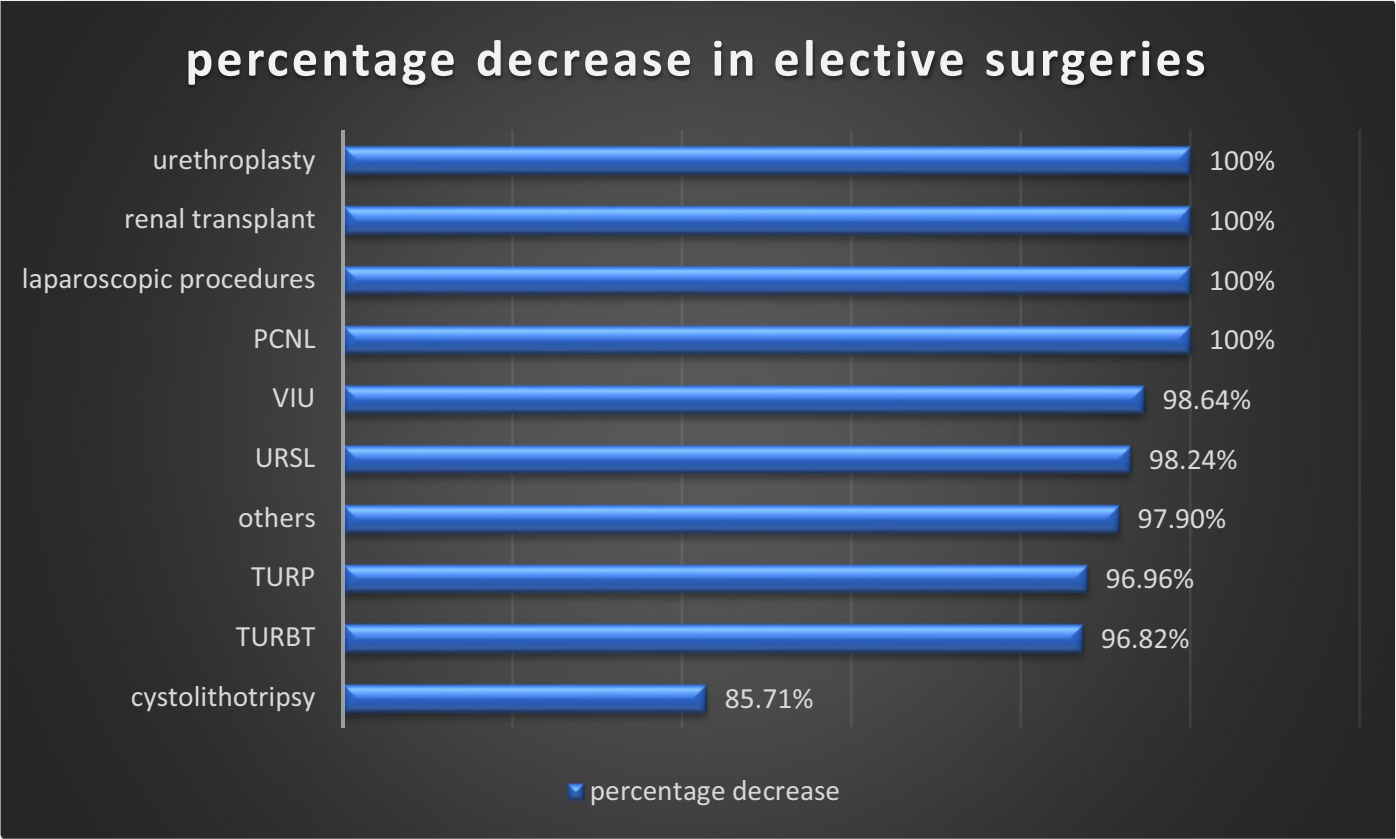
Percentage decrease in elective surgeries during COVID period

There was a significant decrease in all types of elective urological surgeries. In particular, percutaneous nephrolithotomy, ureteroscopic lithotripsy, transurethral resection of the prostate, urethroplasty, and renal transplant had the highest cut-down. Instead of definitive elective procedures, DJ stenting and exchange were done in patients with renal and ureteric calculus disease with intractable pain, obstruction, and infection. Patients with BEP and urethral stricture were managed with perurethral catheter and suprapubic catheter change, respectively. Laparoscopic surgeries were strictly avoided due to the risk of transmission among healthcare workers (Fig. 5 and Table 4).

**Table 4:** Percentage decrease in elective surgeries during COVID-19 lockdown

OT name	No. (pre-lockdown)	No. (lockdown)	Percentage decrease
PCNL	374	0	100
URSL	114	2	98.24
TURBT	63	2	96.82
TURP	66	2	96.96
Cystolithotripsy	35	5	85.71
VIU	74	1	98.64
Laparoscopic procedures	86	0	100
Others	381	8	97.90

### Presentations of patients in outpatient clinics

**Fig. 6:**
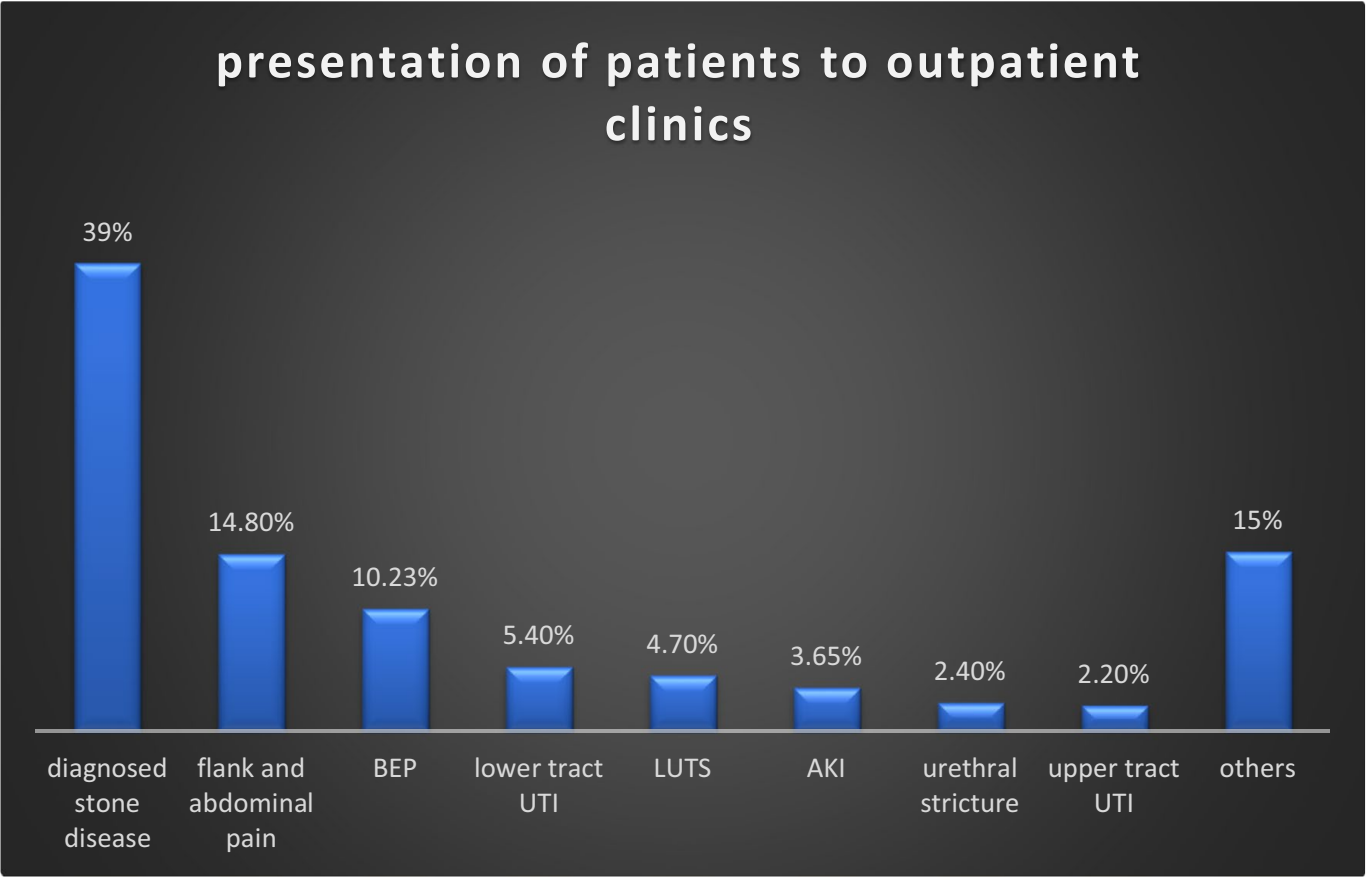
Percentage distribution according to presentation in the outpatient clinic during COVID period

**Fig. 7:**
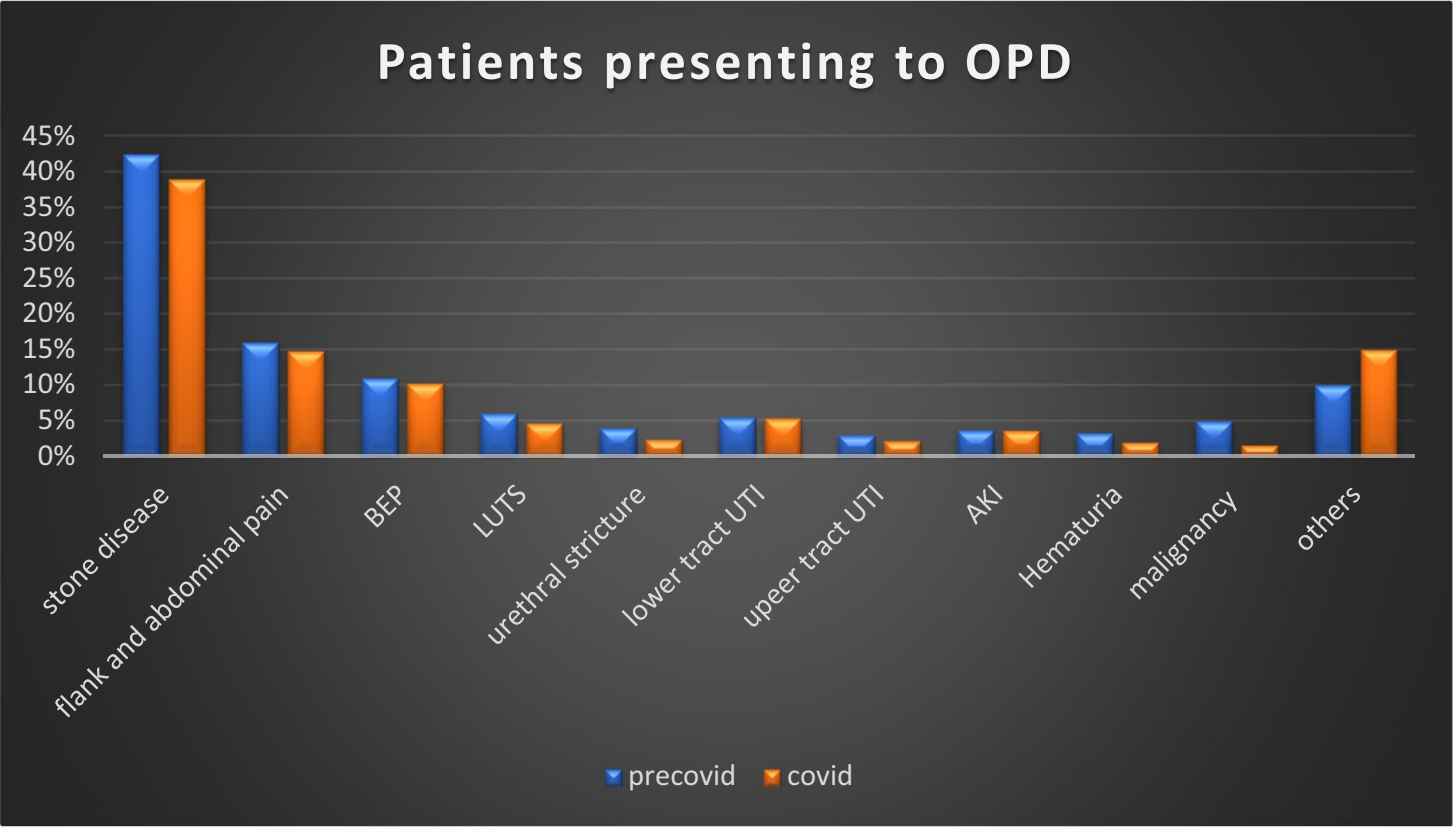
Comparison of patients attending OPD during COVID and pre-COVID period

Previous outpatient attendance in these hospitals was approximately 3000 patients per month, which was reduced to 1260 patients during four months of the lockdown. Overall, there was more than 80% reduction in patients visiting outpatient clinics in public hospitals. Stone disease and its consequences were the main reason for visiting outdoor clinics (39%). A substantial number of patients presented with flank and abdominal pain (14.8%), benign enlargement of the prostate (10.23%), lower urinary tract symptoms (4.7%), and urinary tract infections (5.4%). Malignancy accounted for a very small number of patients visiting outdoor clinics (1.58%) (Figs. 6, 7 and Table 5).

**Table 5:** Distribution of patients attending outpatient clinics

Diagnosis	No
Flank and abdominal pain	187
Diagnosed stone disease	492
BEP	129
LUTS	60
Acute urinary retention	33
Haematuria	25
AKI	46
EPN	8
Pyelonephritis	20
Lower urinary tract infection	69
RCC	3
Ca bladder	8
Urethral stricture	31
Carcinoma prostate	8
Carcinoma penis	1
Angiomyolipoma	2
Other	138
Total	1260

## Discussion

### Effect on indoor admissions, operative procedures, and outpatient clinics

There was a significant decrease in the number of patients presenting to MCGM public healthcare set-up during the lockdown. The reasons attributed to lockdown, lack of transport, fear of coming to COVID-19 public hospitals, and negative image of public hospitals in local media. The majority of patients who came to hospitals were in the age group of 51 to 60 years and were at higher risk for complications if they got infected with COVID-19 [5]. Patients presented mainly due to complications arising from a benign condition. There was a reduction in patients with malignancy presenting in public hospitals. Malignancy accounted for only 9.7% of indoor admissions and 1.58% of the outpatient clinic visits.

There was a significant decrease in indoor admissions and elective operative procedures. As per guidelines issued by both the European Association of Urology (EAU) and the Urology Society of India (USI), stratification of various urological procedures was done. Elective operative procedures for renal and ureteric stones, benign enlargement of the prostate, urethral stricture disease, and renal transplant were affected the most [3, 4]. Semi-emergency procedures like percutaneous nephrostomy insertion, DJ stent insertion and exchange, DJ stent removal, and catheter change were less affected. Patients presenting with urological injuries related to obstetrics and gynaecological procedures had the least decrease in number as they were given priority for transport, diagnosis, and management. Studies done by Devana SK et al., Teoh JY et al., Prasad N et al. showed similar reductions in various urological procedures [6–8]. Similar findings were noted with regard to patients presenting to outpatient clinics. Stone disease and its complications remain the main reason for visiting outdoor clinics (39%). Substantial patients presented with flank and abdominal pain (14.8%), benign enlargement of the prostate (10.23%), lower urinary tract symptoms, urinary tract infections. Malignancy accounted for a very small number of patients visiting outdoor clinics (1.58%).

Our approach and management protocols changed because of COVID-19. Lei S et al. studied the patients undergoing surgeries during COVID-19 and concluded that there was 20% perioperative mortality in COVID-19 carriers and patients. So, there was a necessity to diagnose carriers of COVID-19 before surgery [9]. However, during the earlier period of lockdown nasal and throat swab RT-PCR and CT scan of the chest were not allowed for asymptomatic and planned operative urology patients due to the limited supply of testing kits. Patients with planned surgery were screened by checklist based on travel history, symptoms, and place of stay (zones), and those who were suspected of having COVID-19 underwent RT-PCR. This was the scenario till late June. Thereafter, RT-PCR and CT scan were easily available and used for screening of indoor patients with no or minimal symptoms of COVID-19 [10].

Most of the urology procedures were done in regional or local anaesthesia. General anaesthesia was avoided due to the risk of aerosol generation and transmission of COVID-19. OT protocols were changed, and donning and doffing areas were set up to reduce the risk of transmission to healthcare personal.

Most elective urological surgeries were postponed including endourological stone surgery, prostate surgeries, reconstructive surgeries, incontinence surgeries, infertility and erectile dysfunction surgeries, and genitourinary prolapse surgeries [11, 12]. Urinary diversion, treatment of sepsis, supportive treatment, and care were the cornerstone of management. Percutaneous nephrostomy and DJ stenting for the obstructive stone disease were initially attempted under local anaesthesia and if necessary regional or general anaesthesia was given. Acute urinary retention was managed by the insertion of a urethral or suprapubic catheter under local anaesthesia. In case of clot retention due to bladder cancer, prostate cancer, or benign enlargement of the prostate cystoscopy clot evacuation and haemostasis were performed. Definitive surgery was postponed for 2–3 months. Neoadjuvant chemotherapy was given to patients with malignancy as indicated. Patients who were scheduled for Radical Prostatectomy were given GnRH analogue. As per guidelines of EAU, BAUS, USANZ, radical prostatectomy for low-risk and intermediate-risk prostate cancer was postponed for 3 months. Children and patients with malignancy, trauma, obstetric, and gynaecological emergencies were given priority for treatment.

As per guidelines recommended by the Urology Society of India, laparoscopy and robotic procedures were avoided due to the risk of aerosol transmission [13, 14]. The live renal transplant program was temporarily suspended in all public hospitals as per MoHFW’s Advisory for Hospitals and Medical Institutions dated 03-03-2020 [15]. The long-term impact of delay in urological procedures will require further studies.

### Effect on the training of residents

All urology residents were posted for COVID-19-related duties in rotation which affected the training of residents in routine and emergency urological surgeries. A nationwide survey of the impact of COVID-19 on urology residency in India by Cheriyan A. and Kumar S. concluded that there is a 90% reduction in caseload and surgical exposure leading to a negative impact on surgical training [16]. Effect on academic learning was less profound as online webinars and teaching sessions were increased as compared to pre-COVID era, and the writing of scientific project papers and textbooks was done more frequently.

## Conclusions

COVID-19 pandemic has a profound impact on patient care and education in urology. There was more than ninety percent reduction in indoor admissions, operative procedures, and outpatient clinics attendance. Stone disease was the most common indication for indoor admission (53.65%) and outpatient clinic visits (39%). Malignancy accounted for 9.75% of indoor admissions and 1.58% of outpatient clinics visit. DJ stenting was the most common operative procedure performed. Once the pandemic is controlled, there will be a large number of patients seeking consultation and management for urological conditions and we should be prepared for it. Surgical training of urology residents needs to be compensated in near future. Long-term impact on urological patient outcome remains to be defined.

## Data Availability

The datasets used and/or analysed during the current study are available from the corresponding author on reasonable request.

## Abbreviations

COVID-19: Coronavirus disease
MCGM: Municipal Corporation of Greater Mumbai
DJ: Double J
PCN: Percutaneous nephrostomy
EPN: Emphysematous pyelonephritis
PUC: Perurethral catheter
SPC: Suprapubic catheter
TURBT: Transurethral resection of bladder tumour
VIU: Visual internal urethrotomy
TURP: Transurethral resection of the prostate
PCNL: Percutaneous nephrolithotomy
URSL: Ureterorenoscopic lithotripsy
LUTS: Lower urinary tract symptoms
RT-PCR: Reverse transcriptase polymerase chain reaction
